# Content of Heavy Metal in the Dust of Leisure Squares and Its Health Risk Assessment—A Case Study of Yanta District in Xi’an

**DOI:** 10.3390/ijerph15030394

**Published:** 2018-02-25

**Authors:** Tianjie Shao, Lihuan Pan, Zhiqing Chen, Ruiyuan Wang, Wenjing Li, Qing Qin, Yuran He

**Affiliations:** 1 School of Geography and tourism, Shaanxi Normal University, Xi’an 710062, China; lihuanpan@126.com (L.P.); wangry@snnu.edu.cn (R.W.); wenjing642@163.com (W.L.); 15771936008@163.com (Q.Q.); 15929902657@163.com (Y.H.); 2 SNNU-JSU Joint Research Center for Nanoenvironment Science and Health, Shaanxi Normal University, Xi’an 710062, China

**Keywords:** leisure square, dust, heavy metal, health risk assessment

## Abstract

Taking Yanta District in Xi’an as the research object, the present study measures the contents of Cadmium (Cd), Lead (Pb), Copper (Cu), Nickel (Ni), and Chromium (Cr) in dust samples and further assesses the health risk of heavy metals intake through dust based on the assessment method of human exposure risk proposed by U.S. EPA, with an aim to investigate the content of heavy metal in the dust of leisure squares and its exposure risk. As the results indicate, the average contents of five heavy metals are obviously higher than the soil background value in Shaanxi Province. Therefore, Cd, Ni, Cu, Pb, and Cr are obviously enriched in urban surface dust in Shaanxi Province, due to the influence of human activities. In addition, it can also be found that the non-carcinogen exposure risk in children is significantly higher than that in adults with the risk values of these five heavy metals all one order of magnitude higher than those of adults. Irrespective of whether addressing the results for children or adults, the non-carcinogen exposure doses of five heavy metals are sorted as Cr > Pb > Cu > Ni > Cd. According to the present situation, for a child, the total non-carcinogenic risk values of five heavy metals have exceeded the safety limit in 11 of the 20 leisure squares in Yanta District of Xi’an. That means the leisure squares are no longer suitable for physical and recreational activities. For the five heavy metals, the average non-carcinogenic risk value of Cr is largest, and causes the largest threat to health in Yanta District, Xi’an. The carcinogenic exposure doses of the heavy metals Cr, Cd, and Ni are very low in respiratory pathways and there is no carcinogenic health risk. In general, the Cr content in dust in domestic cities is higher than that of foreign cities; however, the Pb content is much lower.

## 1. Introduction

Owing to frequent human activities, such as transportation, social activities, and industrial production, there are more or less heavy metals in urban dust. Generally speaking, heavy metals in dust are mainly ingested through hand-mouth feeding, inhalation, and skin contact, which all will exert an unfavorable influence on human health [1,2,3]. Surface dust has become a main source of pollution in the urban environment. Therefore, much importance should be attached to the heavy metal pollution of dust in urban leisure squares [4,5], since these squares serve as urban public living places, the centers of social life of urban citizens, and the main places for the elderly to exercise and children to play. At present, quite a few studies, both at home and abroad, have been carried out in terms of the aspect of the content of heavy metals in urban dust. For example, Chang Jing has found that the contents of Pb, Zn, Cd, Cu, Ni, and Cr in street dust in Shanghai, respectively, are 49.3~847.1 mg/kg, 322.3~1722.0 mg/kg, 0.51~1.52 mg/kg, 91.5~495.0 mg/kg, 33.7~138.4 mg/kg, and 81.6~331.4 mg/kg, with average values of 264 mg/kg, 687.25 mg/kg, 0.97 mg/kg, 186.41 mg/kg, 64.91 mg/kg, and 218.91 mg/kg [6]. Similar studies have also been done in places like Nanjing [7], Tianjin [8], Shenyang [9], Chengdu [10] and Beijing [11] of China, Queensland [12], Imam Khomeini of Iran [13], Western and Central Bohemia of Czech [14], London, and some other European cities [15]. As for the heavy metal ingredient analysis and their pollution level in urban street dust in China, 3720 urban street dust samples were collected from 39 large and media-sized cities. The results show that the average mass ratios of As, Cd, Cr, Cu, Hg, Ni, Pb, and Zn in urban street dust in China are 17.82 mg/kg, 3.67 mg/kg, 152.85 mg/kg, 143.58 mg/kg, 0.66mg/kg, 46.74 mg/kg, 220.88 mg/kg, and 602.30 mg/kg, respectively. The heavy metal content overall variation amplitude is larger, which exhibits a skewed distribution, among which the variation coefficient of Cd is greater than 100%, which indicates that the mass ratio of Cd in surface dust in different cities in China is greatly affected by anthropogenic sources [16]. On the whole, the average mass ratios of Cd, Hg, and Pb are the largest, and also the most polluted in China [16]. Compared with the data in foreign countries, the ratios of Cd, Hg, and Pb are 4.41, 2.56, and 2.77 times higher than the corresponding values in foreign countries [13,17,18,19,20], indicating the serious heavy metal pollution of urban dust in China.

Based on the facts and data presented above, the present study, taking leisure squares in Yanta District in Xi’an (northwest China) as the research object, probes into the content of heavy metals in dust and its health risk, with an aim to provide theoretical references for the assessment of urban environmental quality and livable function. 

## 2. Samples and Method

### 2.1. Samples Collection and Treatment

On a fine day of February in 2016, dust samples were collected from 20 leisure parks (as is shown in Table 1) with plastic brushes and dustpans. Altogether, there were 20 samples, each of which was made up of five dust mixtures collected from one leisure park via the quincunx layout method, with an average weight of 200 g. Then, the contents of heavy metals in ground dust samples, which were kept in self-sealing bags, remained to be measured. The locations of leisure parks and symbolic representations are shown in Table 1 below. 

Having been taken back to laboratory, the collected dust samples were air-dried and then filtered with a 1mm nylon sieve, in order to remove small stones, leaves, and hair, etc. Then, the dust samples, after being ground by a soil grinder, were sieved to less than 75 µm to remove large soil particles and then preserved in self-sealing bags for the measurement of heavy metal contents.

### 2.2. Analysis of Samples

The samples should be digested with the mixed acid of HF-HNO_3_-HClO_4_. The specific steps were described as follows. Weigh 0.50 g dry samples and put them in the crucible with 50 mL Polytetrafluoroethylene and then add 5 mL HF, 5 mL HNO_3_, and 3 mL HCIO_4_. Heat this crucible to a nearly drying state. Then, add 3 mL HF, 3 mL HNO_3_, and 1 mL HClO_4_ again and heat to a nearly drying state. A total of 5 mL 1 mol/L HNO_3_ was used to dissolve the residue. At last, hold the sample in a 25 mL capacity bottle. The measurement of the contents of Cu, Pb, Zn, Cd, and Ni in leisure squares dust can be achieved by the atomic absorption spectrophotometer (Type ZEEnit 700P, Analytik Jena AG, made in Jena, Germany). 

The sample collection area (Yanta District in Xi’an) is located in the south of the loess plateau, and the loess formation is more than 100 m, which is a typical loess distribution area. Therefore, the heavy metal contents in the dust of leisure squares are closely related to loess. For this purpose, the national standard soil sample (GSS-8, loess) was used for the quality control with the guarantee reagent (GR) regarded as the standard for the analysis process. The recovery rate of the measured metal elements is between 90% and 105%. One reagent blank is required for every 10 samples. The water used in the whole process should be ultrapure water. All the work was done in the environmental science laboratory of Shaanxi Normal University.

### 2.3. Data Analysis and Calculation Method

#### 2.3.1. Calculation of Heavy Metal Exposure

Based on the assessment method of human exposure risk proposed by U.S. EPA, the present study aims to assess the dust exposure risk among residents in Yanta District in Xi’an. In general, all these five kinds of heavy metals have a chronic non-carcinogen risk, among which Cd, Cr, and Ni also carry a carcinogen risk. In the present paper, we suppose that residents in leisure parks inhale the heavy metals in dust mainly through hand-mouth feeding, inhalation, and skin contact. It is generally believed that the non-carcinogen exposure risk can be calculated by adding the risk of different elements in three exposure ways, without taking into consideration the interaction between various metals and human body and the toxic differences of pollutants [21]. Owing to the lack of studies on basic parameters of dust emission characteristics, the present study adopts the evaluation guidelines of a domestic site environment and criteria for soil health assessment proposed by U.S.EPA, for the investigation of parameters such as the uptake rate, particle release, volatile factor, and biological exposure [22,23,24].

In the present study, unit time and unit weight of human body to pollutants are used to express the pollutant exposure [mg/(kg·d)]. Formulae 1~3 denote the daily average dose of dust through hand-mouth feeding, skin contact, and inhalation, respectively. Formula (4) refers to the average daily exposure through carcinogenic heavy metal inhalation. Studies of the available carcinogenic risk parameters in the current assessment standards are only carried out from the perspective of inhalation way, without the carcinogenic exposure reference data of hand-mouth feeding and skin contact. Therefore, only daily average exposure for life through inhalation is taken into consideration in the present study.

The daily average exposure dose through hand-mouth feeding (ADD_ing_):

(1) $${\mathrm{ADD}}_{\mathrm{ing}}=C\times \frac{\mathrm{IngR}\times \mathrm{EF}\times \mathrm{ED}}{\mathrm{BW}\times \mathrm{AT}}\times 10^{-6}$$

The daily average exposure dose through skin contact (ADD_dermal_):

(2) $${\mathrm{ADD}}_{\mathrm{dermal}}=C\times \frac{\mathrm{SA}\times \mathrm{SL}\times \mathrm{ABS}\times \mathrm{EF}\times \mathrm{ED}}{\mathrm{BW}\times \mathrm{AT}}\times 10^{-6}$$

The daily average exposure dose through inhalation (LADD_inh_):

(3) $${\mathrm{ADD}}_{\mathrm{inh}}=C\times \frac{\mathrm{InhR}\times \mathrm{EF}\times \mathrm{ED}}{\mathrm{PEF}\times \mathrm{BW}\times \mathrm{AT}}$$

The daily average exposure for life through inhalation of carcinogenic heavy metal (LADD_inh_):

(4) $${\mathrm{LADD}}_{\mathrm{inh}}=\frac{C\times \mathrm{EF}}{\mathrm{PEF}\times \mathrm{AD}}\times \left(\frac{{\mathrm{InhR}}_{\mathrm{child}}\times {\mathrm{ED}}_{\mathrm{child}}}{{\mathrm{BW}}_{\mathrm{child}}}+\frac{{\mathrm{InhR}}_{\mathrm{adult}}\times {\mathrm{ED}}_{\mathrm{adult}}}{{\mathrm{BW}}_{\mathrm{adult}}}\right)$$

In the formula, ADD_ing_ refers to the daily average exposure of dust particles in the manner of hand-mouth feeding. ADD_inh_ denotes the daily average exposure of dust particles in the manner of inhalation. ADD_dermal_ represents the daily average exposure of dust particles in the manner of skin contact. LADD_inh_ refers to the daily average exposure for life through the inhalation of carcinogenic heavy metal. The values of other parameters in the above formulas are referenced from the U.S. EPA soil health risk assessment method [25], China site environmental assessment guide [24,26], and related domestic and foreign studies [27]. The values of the specific parameters are shown in Table 2.

#### 2.3.2. Calculation of Risk Value of Heavy Metals in Dust

The model used in this study assumes that specific expressions of the carcinogenic and non-carcinogenic risk of heavy metals are given as Formulas (5)–(8), and non-carcinogenic risk is assessed according to the reference dose for chronic poisoning. When the dose in contact with the receptor is within the reference value, it is considered harmless; otherwise, it is at risk. The assessment of the carcinogenic risk of being exposed to street dust can be calculated by average daily exposure in a lifetime.

HQ = ADD/RfD(5)

HI = ∑HQ_i_ (6)

Risk = LADD × SF(7)

Risk_T_ = ∑Risk_i_ (8)

In the formulas, HQ is a non-carcinogenic risk factor that characterizes the non-carcinogenic risk of a single contaminant through a pathway; ADD is a non-carcinogenic risk of a single contaminant from a pathway; RfD is the reference dose for the pathway [24,25], indicating the maximum amount of contaminant absorbed in unit weight and unit time that cannot cause adverse reactions in the body (mg·kg^−1^·d^−1^); HI is a total non-carcinogenic risk for some contaminant in a wide range of exposure ways, and total HI is the sum of all non-carcinogenic risks in all ways. It is generally believed that when HQ or HI < 1, the risk is small or negligible, and when HQ or HI > 1, there is a non-carcinogenic risk; the slope coefficient (SF) reveals the maximum probability of the carcinogenic effect on the human body exposed to a certain dose of some pollutants (mg·kg^−1^·d^−1^) [25]; Risk refers to the risk of cancer, indicating the probability of cancer, usually expressed in the proportion of cancer population in unit population. If Risk is within the range from 10^−6^ to 10^−4^ (that is, one cancer patient in ten thousand to one million people), it is believed that the substance is not provided with the risk of cancer. 

All the data management and statistical analyses in the present study are achieved through SPSS 22 and Excel 2016.

## 3. Results and Discussion

### 3.1. The Content of Heavy Metals in Dust in Leisure Squares in Yanta District in Xi’an

**Figure 1:**
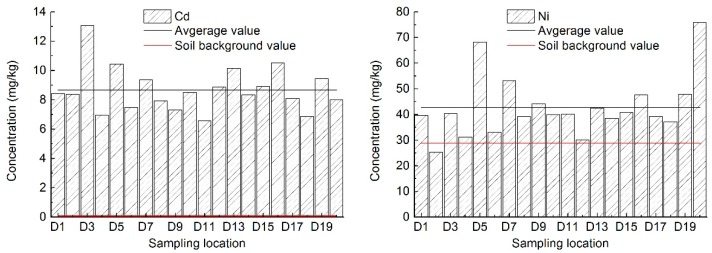

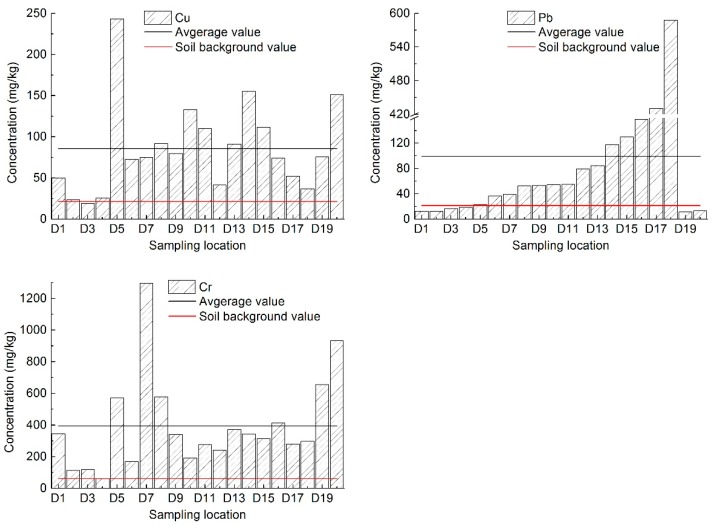
Heavy metal contents in dust in leisure squares of Yanta District of Xi’an.

It is shown in Table 3 and Figure 1 that in the leisure squares of Yanta District in Xi’an, the content ranges of Cd, Ni, Cu, Pb, and Cr are 6.56~13.07 mg/kg, 25.38~75.90 mg/kg, 19.03~243.03 mg/kg, 11.08~588.00 mg/kg, and 60.10~1297.50 mg/kg, respectively, and the average contents are 8.68 mg/kg, 42.74 mg/kg, 85.61 mg/kg, 99.29 mg/kg and 395.57 mg/kg respectively, which are 92.31 times, 1.48 times, 4.00 times, 4.64 times, and 6.33 times higher than soil background values in Shaanxi Province [29]. The ratios of Cd, Ni, Cu, Pb, and Cr in soil that exceed soil background values are 100%, 95%, 95%, 70%, and 95%, respectively. It can be seen that heavy metals such as Cd, Ni, Cu, Pb, and Cr are obviously enriched in road dust due to the influence of human activities. This is especially true for the carcinogenic Cd and Cr, whose mass ratios in road dust are, respectively, 92.31 and 6.33 times bigger than the background concentrations in soil, thereby exerting a relatively pronounced impact on the health of residents. In addition, the quite large standard deviations and coefficients of variation of Pb, Cr, and Cu indicate that the contents of these three elements are not equally distributed at each sampling point, which is due to the differences in the industrial layout and traffic intensity around leisure squares.

It is shown in Figure 1 that in most of the 20 leisure squares in the Yanta District of Xi’an, the contents of heavy metals Cd, Ni, Cu, Pb, and Cr in dust are higher than the soil background value of Shaanxi Province. In the case of Cd elements, its excess multiple is between 69.73 and 139.04 times, and the overall situation is pretty serious. There are five leisure squares with the content of Cd exceeding the standard above 100 times, which are D3 (Zhenguan Square), D5 (Yanxiang Plaza), D13 (Southwest Corner Plaza), D16 (Qide Cultural Plaza), and D19 (High-tech electronic Plaza), respectively. The lowest content of Cd in the 20 leisure squares is D11 (South Plaza of Great Wild Goose Pagoda), and the highest is D3 (Zhenguan Square). For Ni, the excess multiple is between 0.88 to 2.64 times, and its pollution level is relatively slight. The lowest of which is D2 (Fitness Plaza). There are two leisure squares whose Ni contents are two times higher than the standard, which are D5 (Yanxiang Plaza) and D20 (Fountain Square in Shaanxi Normal University). For Cu, its excess multiple is between 0.89 and 11.36 times. There are six leisure squares whose Cu content exceeds the standard above five times, which are D5 (Yanxiang Plaza), D10 (Metro Square), D11 (South Plaza of Great Wild Goose Pagoda), D14 (Jinhong Style Garden), D15 (Ziwei Garden Plaza), and D20 (Fountain Square in Shaanxi Normal University). The lowest of which is D3 (Zhenguan Square), the highest is D5 (Yanxiang Plaza). For Pb, its excess multiple is between 0.52 and 27.48 times, variation amplitude relatively large. The lowest and highest of which are D3 (Zhenguan Square) and D5 (Yanxiang Plaza), respectively. There are two leisure squares whose Pb contents are above 10 times higher than the standard, which are D17 (Martyr Square) and D18 (Mingdemen Community Plaza); in addition, there are six leisure squares which unexceeded that of the standard, which are D1 (Harmony Plaze), D2 (Fitness Plaza), D3 (Zhenguan Square), D4 (Sunken Plaza), D19 (High-tech electronic Plaza), and D20 (Fountain Square in Shaanxi Normal University), respectively. For Cr, its excess multiple is between 0.96 and 20.76 times, and the variation amplitude also relatively large. The lowest and highest of which are D4 (Sunken Plaza) and D7 (Zhonghe Plaza), respectively. There are three leisure squares whose Cr contents are above 10 times higher than the standard, which are D7 (Zhonghe Plaza), D19 (High-tech electronic Plaza), and D20 (Fountain Square in Shaanxi Normal University).

### 3.2. Analysis of Heavy Metal Risk Exposure in Dust of Leisure Squares in Yanta District of Xi’an

The daily average exposure doses and total exposure doses of unit weight in the three ways are calculated with related parameters in Formulas (1)–(3) and Table 2, and the results shown in Table 4 indicate that the average exposure doses of five heavy metals in unit weight of children are almost all one magnitude higher than for adults; whether it is for children or adults, the five heavy metals are sorted as Cr > Pb > Cu > Ni > Cd based on the size of the exposure dose.

### 3.3. Risk Assessment of Non-Carcinogenic Exposure in Leisure Squares of Yanta District in Xi’an

According to RfD in Table 5, the non-carcinogenic reference doses of the five heavy metals and the exposure risk values HQ are calculated in three ways, relating to hand-mouth feeding, inhalation, and skin contact, as presented in Table 6 and Table 7. It can be seen from Table 6 that heavy metal Cr in leisure parks causes the highest non-carcinogenic risk for children, with a non-carcinogenic risk value of 9.19 × 10^−1^, very close to its non-carcinogenic risk threshold; the next is heavy metal Pb with a non-carcinogenic risk value of 1.88 × 10^−1^. Then comes the heavy metal Cd with a non-carcinogenic risk value of 6.36 × 10^−2^. The non-carcinogenic risk values of Cu and Ni are the smallest, with a value of 1.41 × 10^−2^ for both. It is worth mentioning that the superposed non-carcinogenic risk value of all heavy metals ingested in the hand-mouth feeding pathway has exceeded 1. This means that it has been a threat to local people’s health.

For adults, the non-carcinogenic risk of heavy metal Cr in the dust of leisure squares is the highest, with a non-carcinogenic risk value of 1.01 × 10^−1^, followed by heavy metals Pb and Cd with non-carcinogenic risk values of 2.02 × 10^−2^ and 7.03 × 10^−3^, respectively. Non-carcinogenic risk values of heavy metals Cu and Ni are the smallest, with a value of 1.52 × 10^−3^ for both (Table 7). Fortunately, the superposed risk of all heavy metals ingested in all pathways does not exceed 1. In other words, the non-carcinogenic risk of adult is controlled within the safety limit.

Table 6 and Table 7 show that the overall non-carcinogenic risk for children is higher than that for adults. The non-carcinogenic risk values of the five heavy metals in hand-mouth feeding and skin contact pathways are one magnitude higher than those of adults.

**Figure 2:**
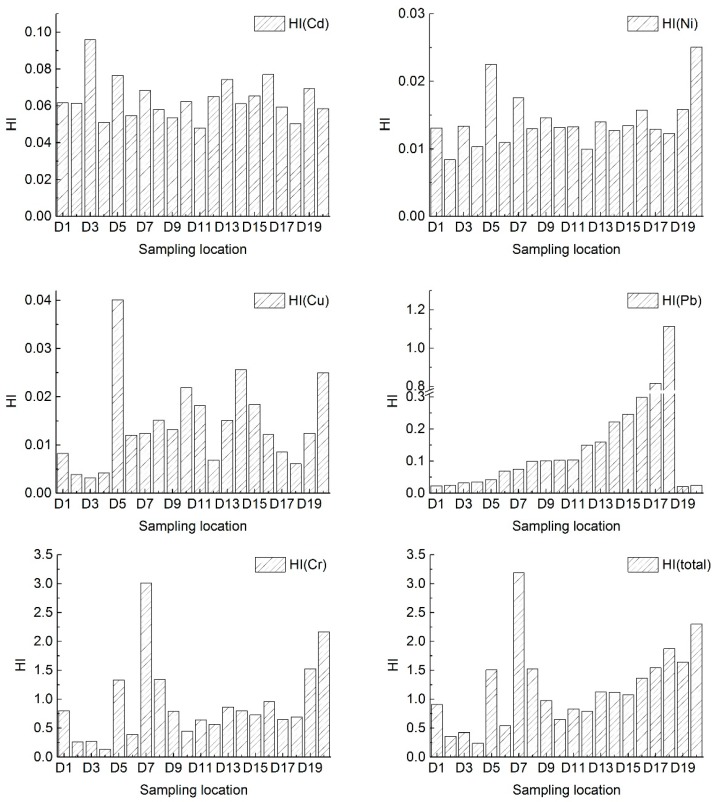
Non-carcinogenic risk value (child) of heavy metal in leisure squares of Yanta District in Xi’an.

When considering that the three ingested ways of hand-mouth feeding, inhalation, and skin contact exist together, the average non-carcinogenic risk values of single heavy metal in leisure squares of Yanta District is less than 1 (Table 6 and Table 7), but it will exceed 1 in some sampling sites. Therefore, we should pay more attention to the associated health risk. This is particularly significant for children, because they are more sensitive to heavy metal pollution. For this purpose, the present study analyzes the non-carcinogenic risk to children in 20 urban leisure squares in Yanta District in Xi’an, and the results are shown in Figure 2. As is illustrated in Figure 2, the total non-carcinogenic risk values (HI (total)) for children in D7 (Zhonghe Plaza) and D20 (Fountain Square in Shaanxi Normal University) are 3.19 and 2.30, respectively, more than twice the safety limit. These are immediately followed by the D18 (Mingdemen Community Plaza), D19 (High-tech electronic Plaza), D17 (Martyr Square), D8 (Kaiyuan Plaza), D5 (Yanxiang Plaza), D16 (Qide Cultural Plaza), D13 (Southwest Corner Plaza), D14 (Jinhong Style Garden), and D15 (Ziwei Garden Plaza), with values of 1.87, 1.64, 1.54, 1.53, 1.51, 1.36, 1.13, 1.12, and 1.07, respectively, also exceeding the safety limit of 1. The total non-carcinogenic risk values (HI (total)) of the other nine leisure squares are below the safety limit. D2 (Fitness Plaza), D3 (Zhenguan Square), and D4 (Sunken Plaza) are all under half of the limit. However, the values of D1 (Harmony Plaze) and D9 (Seasonic Square) are close to the safety limit of 1.

Non-carcinogenic risk values of heavy metal Cr are 3.01, 2.17, 1.52, 1.34, and 1.33 in D7 (Zhonghe Plaza), D20 (Fountain Square in Shaanxi Normal University), D19 (High-tech electronic Plaza), D8 (Kaiyuan Plaza), and D5 (Yanxiang Plaza), respectively, for children, beyond the safety limit of 1, which will result in a negative impact on children’s health; the non-carcinogenic risk value of heavy metal Cr is 0.96 in D16 (Qide Cultural Plaza) for children, close to the safety limit of 1—so it is not suitable for children to stay in these leisure squares. Non-carcinogenic risk values of heavy metal Pb do not exceed the safety limit of 1, except in D18 (Mingdemen Community Plaza) (Figure 2). Non-carcinogenic risk values of heavy metal Cd, Ni, and Cu are small and far lower than the safety limit, which are not harmful to the children’s health. According to the present situation, 14 of the 20 leisure square had been polluted by heavy metal Pb or Cr. There are no longer suitable for children’s activities. Hence, in general, the major heavy metal pollution is Cr and Pb in dust in leisure squares in Yanta district in Xi’an. Therefore, relevant departments should strengthen the prevention and governance of heavy metal Cr and Pb pollution.

### 3.4. Risk Assessment of Carcinogenic Exposure in Leisure Squares of Yanta District in Xi’an

Lifetime average exposure doses of carcinogenic heavy metal Cd, Ni, and Cr in inhalation pathways are calculated according to Formula (4), with the results in Table 8 showing that the three heavy metal elements Cd, Ni, and Cr are sorted as Cr > Cd > Ni according to the size of exposure doses, yet the carcinogenic risk exposure dose is too low to threaten human health.

### 3.5. Comparison of the Dust Heavy Metal Concentrations with Other Cities

**Figure 3:**
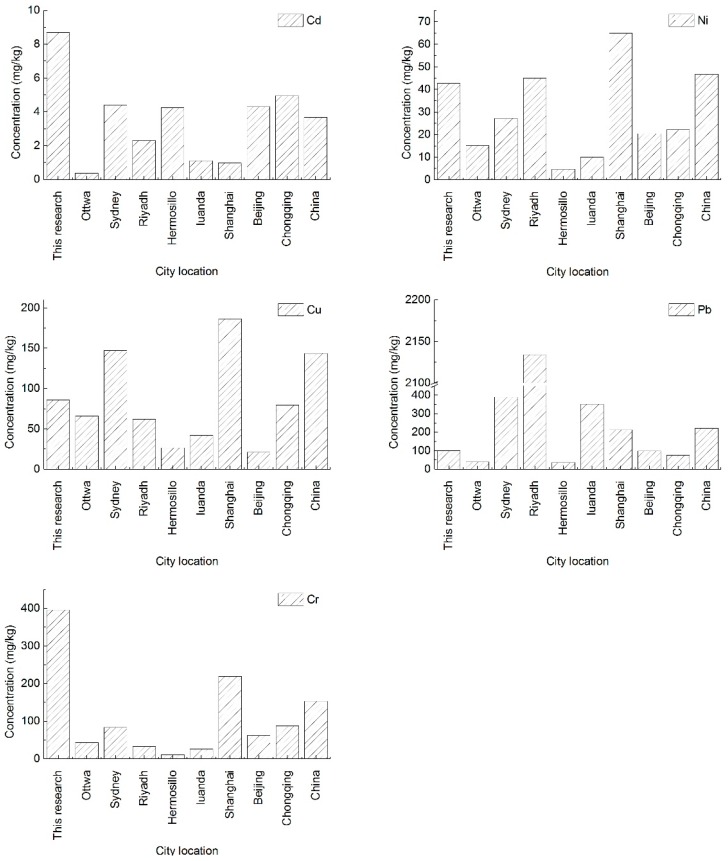
Comparison of the dust heavy metal contents in leisure squares of Yanta District of Xi’an with other cities.

At present, heavy metals in urban road dust such as Cd, Ni, Cu, Pb, and Cr have been mostly studied from the perspective that the enrichment of these elements has important environmental significance and serious environmental pollution hazards [30,31,32]. As shown in Figure 3—the contents of heavy metals in road dust of some cities at home and abroad, the contents of different elements in road dust in different cities are quite different from each other, which is mainly related to the size of the urban population, traffic volume, urban topography, and meteorology, as well as the background concentrations in soils around the country. In terms of the urban leisure squares in Yanta District, Xi’an City, the Cr content in road dust is 2.37 times higher than its average in China [16] and about 1.97–23.46 times higher than that of other cities at home and abroad [6,17,19,27,33,34,35]. The Ni content is similar to the national average and to Riyadh [33], but obviously higher than that of Ottwa [20], Sydney [19], Hermosillo [17], Luanda [27], Beijing [34], and Chongqing [35], etc. The Cu content is below the national average and also Sydney [19] and Shanghai [6], but significantly higher than Ottwa [20], Riyadh [33], Hermosillo [17], Luanda [27], and Beijing [34]. The Pb content is remarkably lower than the national average and Sydney [19], Riyadh [33], Luanda [27], and Shanghai [6], etc., but higher than Ottwa [20], Hermosillo [17], and Chongqing [35], etc. In general, the Cr content in dust in domestic cities is higher than that of foreign cities, which is mainly due to the high background concentration of soil. However, owing to the large car ownership in developed western countries, the Pb content in road dust emitted from car exhausts is much higher than that of domestic cities. It is worth mentioning that although all the selected cities are important cities around the world, and the sample particle sizes are mainly focused on below 75–100 µm, due to the difference of the specific size and sampling area, the results may not completely accurately express the differences between cities. However, there is a rough understanding of the differences between them.

## 4. Conclusions

Based on the analysis and discussion above, conclusions can be drawn as follows.

1. After the measurement of heavy metal contents in dust in 20 leisure squares in Yanta District of Xi’an, it is found that the average contents of five heavy metals are 92.31 times, 1.48 times, 4.00 times, 4.64 times, and 6.33 times significantly higher than the soil background values in Shaanxi Province. The exceeding standard rates of Cd, Ni, Cu, Pb, and Cr are 100%, 95%, 95%, 70%, and 95%, respectively. Therefore, Cd, Ni, Cu, Pb, and Cr are obviously enriched in urban surface dust in Shaanxi Province, due to the influence of human activities.
2. The model of human exposure risk, proposed by U.S.EPA, is employed to assess the health risk. It is found that the average exposure doses and non-carcinogenic risks of the Cd, Pb, Cu, Ni, and Cr are significantly higher than that for adults, and almost all of the average exposure doses and non-carcinogenic risk values of the five heavy metals are one magnitude higher than those for adults. Whether this is for children or adults, the exposure dose of five heavy metals are sorted as Cr > Pb > Cu > Ni > Cd.
3. According to present situation, for children, the total non-carcinogenic risk values of five heavy metals have exceeded the safety limit in 11 of the 20 leisure squares in Yanta District of Xi’an. This means that the leisure squares have been polluted, and are no longer suitable for physical and recreational activities. The remaining nine leisure squares do not exceed 1, that is, the total non-carcinogenic risk is within the limit of safety. Thereinto, the leisure squares with the lowest non-carcinogenic risk are D2 (Fitness Plaza), D3 (Zhenguan Square), and D4 (Sunken Plaza). The average non-carcinogenic risk values of Cr and Pb are largest, of which Pb causes the largest threat to health in Yanta District, Xi’an.
4. The heavy metals in leisure squares in Yanta District of Xi’an are sorted as Cr > Cd > Ni by the possibility of carcinogenic risk in respiratory pathways, but the exposure doses of carcinogenic risk of the three heavy metals are very low and there is no carcinogenic health risk.
5. In general, the Cr content in dust in domestic cities is higher than that of foreign cities, which is mainly due to the high background concentration of soil. However, owing to the large car ownership in developed western countries, the Pb content in road dust emitted from car exhausts is much higher than that of domestic cities.

## Figures and Tables

**Table 1:** Sampling sites and symbolic representations.

Number	Sampling Sites	Number	Sampling Sites
D1	Harmony Plaze	D11	South Plaza of Great Wild Goose Pagoda
D2	Fitness Plaza	D12	North Plaza of Great Wild Goose Pagoda
D3	Zhenguan Square	D13	Southwest Corner Plaza
D4	Sunken Plaza	D14	Jinhong Style Garden
D5	Yanxiang Plaza	D15	Ziwei Garden Plaza
D6	Chongyang Plaza	D16	Qide Cultural Plaza
D7	Zhonghe Plaza	D17	Martyr Square
D8	Kaiyuan Plaza	D18	Mingdemen Community Plaza
D9	Seasonic Square	D19	High-tech electronic Plaza
D10	Metro Square	D20	Fountain Square in Shaanxi Normal University

**Table 2:** Calculation parameter values of the daily average exposure of heavy metals.

Items	Parameters/Unit	Physics Meaning	Values	Data Sources
Basic parameters	C/mg·kg^−1^	concentration of heavy metal	95% UCL	this present study
Exposure behavior parameters	EF/d·a^−1^	exposure frequency	180	[27]
	ED/a	exposure time	6 (child), 24 (adult)	[25,26,27]
	BW/kg	weight per capita	15(child), 70(adult)	[25,26,27]
	AT/d	mean exposure time	ED × 365 (non-carcinogen), 70 × 365 (carcinogen)	[25,26,27]
Hand-Mouth feeding	IngR/mg·d^−1^	Hand-mouth feeding frequency	200 (child), 100 (adult)	[25,26,27]
Skin contact	ABS/non-dimensional	Skin absorption factor	1 × 10^−3^	[25,26,27]
	SA/cm^2^	Surface area of skin exposure	1150 (child), 2145 (adult)	[28]
	SL/mg·cm^−2^·d^−1^	Skin adhesive capacity	0.2 (child), 0.07 (adult)	[27]
Inhalation	InhR/m^3^·d^−1^	respiratory frequency	5.71 (child), 19.02 (adult)	[27]
	PEF/m^3^·kg^−1^	Particulate emission factor	1.36 × 10^9^	[25,26,27]

Note: 95% UCL is in the 95% confidence limit of the average.

**Table 3:** Basic statistical parameters of the mass ratio of heavy metals in dust of leisure squares in Yanta District in Xi’an (mg/kg).

Element	Min.	Max.	Average Value	Standard Deviation	Coefficient of Variation	Soil Background Value	Certified Value	Exceeding Standard Rate (%)
Cd	6.56	13.07	8.68	1.50	0.17	0.094	0.13 ± 0.02	100
Ni	25.38	75.90	42.74	11.63	0.27	28.80	31.5 ± 1.8	95
Cu	19.03	243.03	85.61	53.24	0.62	21.40	24.3 ± 1.2	95
Pb	11.08	588.00	99.29	144.77	1.46	21.40	21.0 ± 2.0	70
Cr	60.10	1297.50	395.57	289.05	0.73	62.50	68.0 ± 6.0	95

**Table 4:** Exposure doses of heavy metals in the dust of urban leisure squares in different ways.

Element	ADD_ing_		ADD_inh_		ADD_derm_		ADD_total_	
	Child	Adult	Child	Adult	Child	Adult	Child	Adult
Cd	5.71 × 10^−5^	6.11 × 10^−6^	1.20 × 10^−9^	8.55 × 10^−10^	6.56 × 10^−8^	9.18 × 10^−9^	5.71 × 10^−5^	6.12 × 10^−6^
Ni	2.81 × 10^−4^	3.01 × 10^−5^	5.90 × 10^−9^	4.21 × 10^−9^	3.23 × 10^−7^	4.52 × 10^−8^	2.81 × 10^−4^	3.02 × 10^−5^
Cu	5.63 × 10^−4^	6.03 × 10^−5^	1.18 × 10^−8^	8.43 × 10^−9^	6.47 × 10^−7^	9.06 × 10^−8^	5.64 × 10^−4^	6.04 × 10^−5^
Pb	6.53 × 10^−4^	6.99 × 10^−5^	1.37 × 10^−8^	9.78 × 10^−9^	7.51 × 10^−7^	1.05 × 10^−7^	6.54 × 10^−4^	7.01 × 10^−5^
Cr	2.60 × 10^−3^	2.79 × 10^−4^	5.46 × 10^−8^	3.90 × 10^−8^	2.99 × 10^−6^	4.18 × 10^−7^	2.60 × 10^−3^	2.79 × 10^−4^

**Table 5:** Non-carcinogenic exposure reference doses of heavy metal in different exposure pathways in Leisure Squares of Yanta District in Xi’an (mg·kg^−1^·d^−1^).

Population	Element	RfD_ing_	RfD_inh_	RfD_derm_
Child &adult	Cd	1.00 × 10^−3^	1.00 × 10^−3^	1.00 × 10^−5^
	Ni	2.00 × 10^−2^	2.06 × 10^−2^	5.40 × 10^−3^
	Cu	4.00 × 10^−2^	4.02 × 10^−2^	1.20 × 10^−2^
	Pb	3.50 × 10^−3^	3.52 × 10^−3^	5.25 × 10^−4^
	Cr	3.00 × 10^−3^	2.86 × 10^−5^	6.00 × 10^−5^

**Table 6:** Non-carcinogenic exposure risk values (child) of heavy metal in different exposure pathways in Leisure Squares of Yanta District in Xi’an.

Element	HQ_ing_	HQ_inh_	HQ_derm_	HI
Cd	5.71 × 10^−2^	1.20 × 10^−6^	6.56 × 10^−3^	6.36 × 10^−2^
Ni	1.40 × 10^−2^	2.86 × 10^−7^	5.98 × 10^−5^	1.41 × 10^−2^
Cu	1.41 × 10^−2^	2.94 × 10^−7^	5.39 × 10^−5^	1.41 × 10^−2^
Pb	1.87 × 10^−1^	3.89 × 10^−6^	1.43 × 10^−3^	1.88 × 10^−1^
Cr	8.67 × 10^−1^	1.91 × 10^−3^	4.99 × 10^−2^	9.19 × 10^−1^
Total	1.14	1.91 × 10^−3^	5.80 × 10^−2^	1.20

**Table 7:** Non-carcinogenic exposure risk values (adult) of heavy metal in different exposure pathway in Leisure Squares of Yanta District in Xi’an.

Element	HQ_ing_	HQ_inh_	HQ_derm_	HI
Cd	6.11 × 10^−3^	8.55 × 10^−7^	9.18 × 10^−4^	7.03 × 10^−3^
Ni	1.51 × 10^−3^	2.04 × 10^−7^	8.37 × 10^−6^	1.52 × 10^−3^
Cu	1.51 × 10^−3^	2.10 × 10^−7^	7.55 × 10^−6^	1.52 × 10^−3^
Pb	2.00 × 10^−2^	2.78 × 10^−6^	2.00 × 10^−4^	2.02 × 10^−2^
Cr	9.29 × 10^−2^	1.36 × 10^−3^	6.97 × 10^−3^	1.01 × 10^−1^
Total	1.22 × 10^−1^	1.37 × 10^−3^	8.11 × 10^−3^	1.31 × 10^−1^

**Table 8:** Carcinogenic risk values of heavy metal in inhalation exposure pathway in Leisure Squares of Yanta District in Xi’an.

Element	LADD_inh_	SF_inh_/(mg·kg^−1^·d^−1^)	Exposure Risk
Cd	2.49 × 10^−9^	6.30	1.57 × 10^−8^
Ni	1.64 × 10^−9^	0.84	1.38 × 10^−9^
Cr	7.58 × 10^−7^	42.00	3.18 × 10^−5^
